# Low ppm NO_2_ detection through advanced ultrasensitive copper oxide gas sensor

**DOI:** 10.1186/s11671-024-04039-z

**Published:** 2024-06-24

**Authors:** Smriti Sihag, Rita Dahiya, Suman Rani, Priyanka Berwal, Anushree Jatrana, Avnish Kumar Sisodiya, Ashutosh Sharma, Vinay Kumar

**Affiliations:** 1 Department of Physics, COBS&H, CCS Haryana Agricultural University, Hisar, Haryana 125004 India; 2 Department of Chemistry, COBS&H, CCS Haryana Agricultural University, Hisar, Haryana 125004 India; 3 Department of Physics, Ramjas College, University of Delhi, Delhi, 110007 India; 4 Department of Material Science and Engineering, Ajou University, Yeongtong-gu, Suwon, 16499 Korea; 5 Amity Institute of Applied Sciences, Amity University, Jharkhand, Ranchi 834002 India

**Keywords:** NO_2_, Precipitation, Copper oxide, Gas-sensor, Low detection limit

## Abstract

The imperative development of a cutting-edge environmental gas sensor is essential to proficiently monitor and detect hazardous gases, ensuring comprehensive safety and awareness. Nanostructures developed from metal oxides are emerging as promising candidates for achieving superior performance in gas sensors. NO_2_ is one of the toxic gases that affects people as well as the environment so its detection is crucial. The present study investigates the gas sensing capability of copper oxide-based sensor for 5 ppm of NO_2_ gas at 100 °C. The sensing material was synthesized using a facile precipitation method and characterized by XRD, FE-SEM, UV–visible spectroscopy, photoluminescence spectroscopy, XPS and BET techniques. The developed material shows a response equal to 67.1% at optimal temperature towards 5 ppm NO_2_ gas. The sensor demonstrated an impressive detection limit of 300 ppb, along with a commendable percentage response of 5.2%. Under optimized conditions, the synthesized material demonstrated its high selectivity, as evidenced by the highest percentage response recorded for NO_2_ gas among NO_2_, NH_3_, CO, CO_2_ and H_2_S.

**Supplementary Information:**

The online version contains supplementary material available at 10.1186/s11671-024-04039-z.

## Introduction

The sensing of hazardous gases is crucial to environmental safety. Due to fast industrialization and increasing numbers of vehicles such gases have become prevalent and pose a serious threat to both human well-being and the environment unless they are efficiently sensed and eradicated [1]. Hazardous gases include nitrogen dioxide (NO_2_), methanol (CH_3_OH), carbon dioxide (CO_2_), nitrous oxide (N_2_O), carbon monoxide (CO), ammonia (NH_3_), hydrogen sulphide (H_2_S), ethanol (CH_3_CH_2_OH), formaldehyde (HCHO) etc. [2–5]. Amongst these gases, nitrogen dioxide is an extremely hazardous and detrimental gas that is mainly produced by industries and automobiles [6].

Even the tiniest amount of NO_2_ (around 1 ppm) can cause adverse effects on our respiratory system, resulting in severe lung conditions that are similar to asthma symptoms [7–9] The NO_2_ has a negative impact on those suffering from chronic obstructive pulmonary disease (COPD) and is regarded as one of the main elements that contribute to this condition [10, 11]. In addition to ozone formation, NO_2_ also negatively impacts both terrestrial and aquatic ecosystems. Photochemical smog, acid rain and eutrophication of coastal waterways like the Chesapeake Bay are all directly caused by the presence of NO_2_ in the environment [12, 13]. So, it is of utmost importance to monitor the NO_2_ and develop a cost-effective and selective sensor that can efficiently detect even very low concentrations of NO_2_ gas while remaining cost-effective and selective. Metal oxides are currently being extensively researched in the gas-sensing field because of their semiconducting properties, high sensitivity and ease of measurement [14]. Although there have been advancements in gas detection technology but many commercial products still rely on n-type semiconductors sensing materials like ZnO, In_2_O_3_, Fe_2_O_3_, SnO2, TiO_2_ and WO_3_ for detecting hazardous gases [15, 16]. The information on application of p-type metal oxide semiconducting materials for gas-sensing is limited [17]. The p-type metal oxide gas sensor has advantages over the n-type metal oxide gas sensor and it also offers a great deal of potential for use in real time applications [18]. There have been reports indicating that p-type metal oxides can readily exchange their lattice oxygen with air for maintaining their stoichiometry [19] and they rely less on high temperatures for conduction. These properties may be exploited to use p-type materials as efficient and long-term usable gas sensors [20]. The copper oxide (CuO) is a highly significant p-type semiconductor with many fascinating properties, including direct bandgap, non-toxic nature, excellent semiconducting ability, economic synthesis, easy availability and environment friendly nature [21–24]. The CuO has a monoclinic crystal system and narrow band gap in the range of 1.2–1.8 eV [25]. It has an extensive range of uses in the areas of photocatalytic activity [26], solar cells [27], batteries [28], photocatalysis [29], electronics [30] and sensors [31] as well. The gas-sensing capabilities of CuO have been the subject of a number of studies. Using the sol–gel process, Wang et al. (2016) produced CuO nanoparticles and investigated their gas-sensing behaviour for 10 ppm of ethanol, methanol and acetone at 220 °C [32]. Wu et al. (2017) synthesized the different morphology of CuO of which spherical structure shows maximum response for 100 ppm of triethylamine at 230 °C [33]. Nakate et al. (2018) synthesized CuO from a chemical route having nano-bitter gourd like structure and investigates its hydrogen gas sensing properties. For 100 ppm of gas response of 175% was recorded at 200 °C and lowest concentration of gas detected 2 ppm [17]. Though some researchers reported work on CuO-based gas sensors, but most of them have low value of the response, high detection limit or high operating temperature. So, there is a continuing requirement for a more efficient, economical and simple approach to develop CuO nanostructures that have superior gas sensing characteristics.

In the work, copper oxide nanostructures were synthesized using a facile precipitation method. Various characterisation techniques were used to examine the structural, surface morphological and optical characteristics of the synthesized material. By drop-casting the synthesised material on a glass substrate, an effective, inexpensive and simple gas sensing material was developed. The gas-sensing properties of the film were studied at different temperatures and for various concentrations of nitrogen dioxide gas. In the last section mechanism behind the efficient detection of NO_2_ gas by copper oxide has been described.

## Experimental section

### Chemicals used

All the chemicals used for synthesis were of analytical grade. For the synthesis of copper oxide, copper nitrate trihydrate Cu(NO_3_)_2_·3H_2_O and pellets of sodium hydroxide (NaOH) were used. Silver paste (99.99% purity) was used for making contacts on the sensor surface. The ethanol was used for cleaning of substrate. All the chemicals were bought from Sigma Aldrich. Throughout the experiment, double-distilled water was utilized.

### Synthesis of copper oxide

**Fig. 1:**
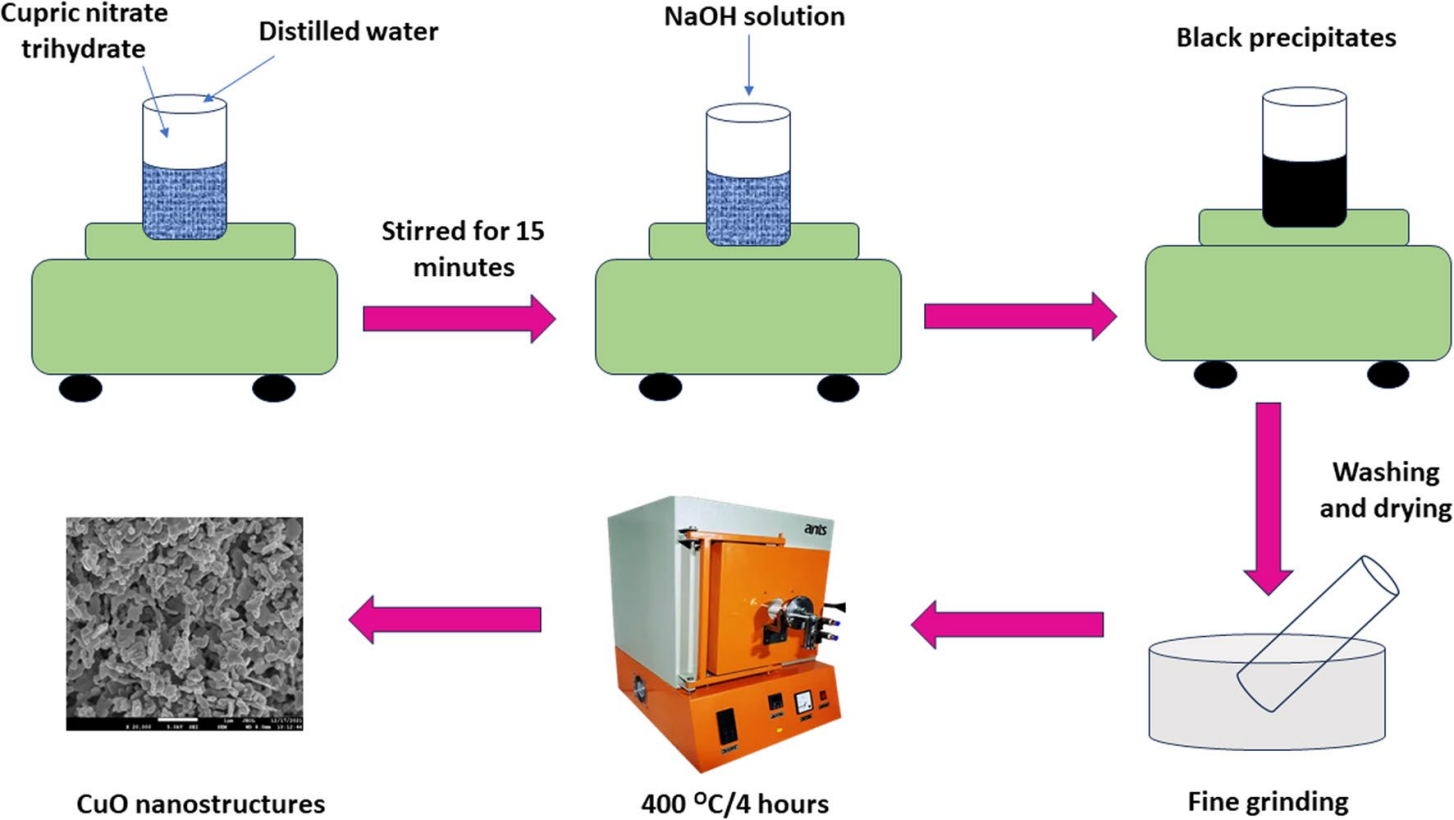
The schematic process representing the synthesis of CuO sample

For the synthesis of copper oxide, precipitation approach was employed [34]. A precise quantity of copper nitrate trihydrate was dissolved in 100 mL of distilled water to get a 0.1 M solution and a 0.5 M solution of sodium hydroxide was prepared. The NaOH solution was dropped into the copper nitrate trihydrate solution with a constant stirring till the black precipitates were obtained. The precipitates were collected after the solution had been left undisturbed for a whole night and the solution was then washed with distilled water. The obtained precipitates were dried at 90 °C and then calcinated at 400 °C for four hours. The schematic process for the synthesis of CuO is shown in Fig. 1.

### Characterization

To analyse the structural properties of the produced CuO, X-ray diffraction (XRD) was conducted with the help of Rigaku Miniflex-II diffractometer using CuKα radiation having wavelength 1.54 Å. The morphological analysis was performed by using field emission scanning electron microscope (FE-SEM), (model number 7610F Plus/JEOL). A UV-2600i spectrophotometer was employed to investigate the optical properties of the synthesized material. Photoluminescence spectrum of the sample was recorded with the help of Luma 40 spectrophotometer. X-ray photoelectron spectroscopy (XPS) was employed to evaluate the chemical states of the elements presented in the sample. The specific surface area and pore size distribution of synthesized material was studied using the BET technique.

### Sensor fabrication and measurements

**Fig. 2:**
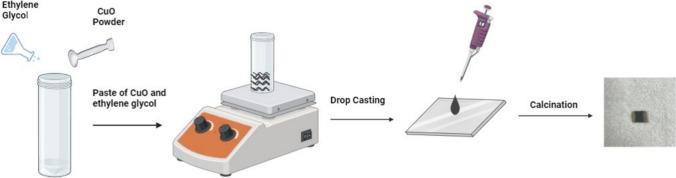
The Schematic diagram outlining the process of drop caste to prepare the sensing film

**Fig. 3:**
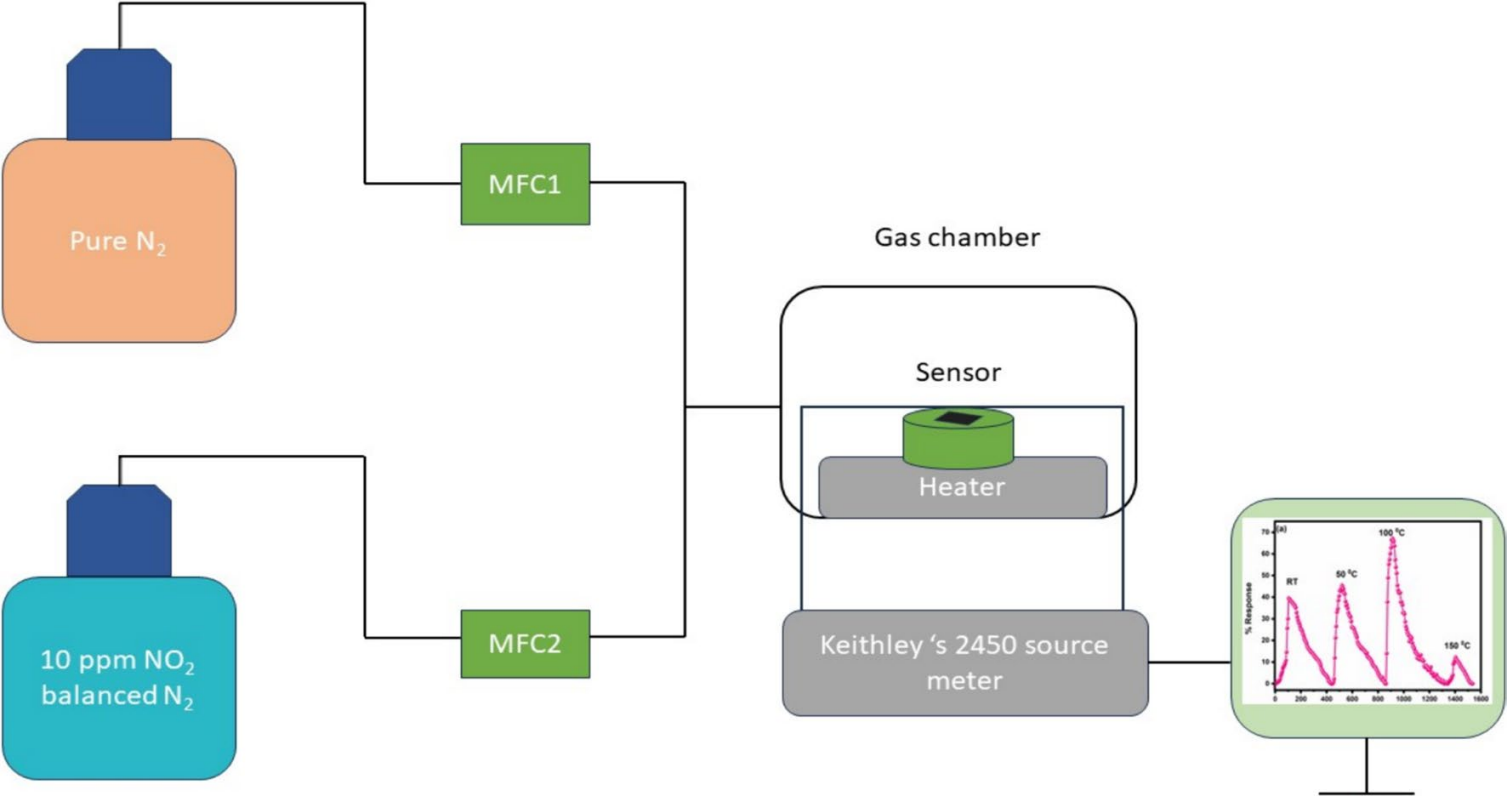
The schematic diagram depicting the gas sensing measurement system

The drop-casting process was used to fabricate the sensing film. A mixture of 25 mg of calcinated powder of synthesized copper oxide and 1 mL of ethylene glycol was made. The mixture was stirred until it was evenly distributed. The resultant paste was drop-cast onto a 1 cm^2^ glass substrate and dried for one hour at 80 °C. The resulting film was then calcined for four hours at a ramp rate of 20 °C in a muffle furnace at 450 °C. For making electrical contacts, silver paste was used. A schematic diagram for the fabrication of the sensing film is shown in Fig. 2. As developed sensing film of CuO was placed inside the testing chamber and change in its current on exposure to gas was noted as a function of time with the help of Keithley 2450 sourcemeter. Figure 3 shows the schematic of the gas sensing measurement system. The gas sensing measurement system consist of custom-made dynamic set up with sensing chamber made of stainless-steel having volume equal to 200 cm^3^ equipped with electric heater. All the gas sensing measurements were measured in-situ with the help of two probe technique using the source meter. Mixed ratio of high purity analyte gases (99.99%) and nitrogen gas (99.99%) was injected inside the chamber at a constant flow rate of 100 sccm which was controlled with the help of two mass flow controllers (MFCs). The total flow rate of analyte gas and nitrogen gas was kept constant when gas concentration was changed. Also, a low vacuum was created in the chamber before each measurement for evacuation and cleaning purposes using a rotary pump provided in the system. The LABVIEW software is used for the measurements of gas sensing parameters. The percentage response (R%) to the target gas was calculated using the following formula,

1 $$R\% = \frac{{I_{a} - I_{g} \times 100}}{{I_{a} }}$$

where I_a_ and I_g_ stands for the currents in the presence of air and target gas, respectively [4]. The time taken to reach 90% of the steady value of the % response was taken as response time, while the time taken to recover to 10% of the initial % response was defined as recovery time [35, 36].

## Results and discussions

### Structural, morphological and optical properties

**Fig. 4:**
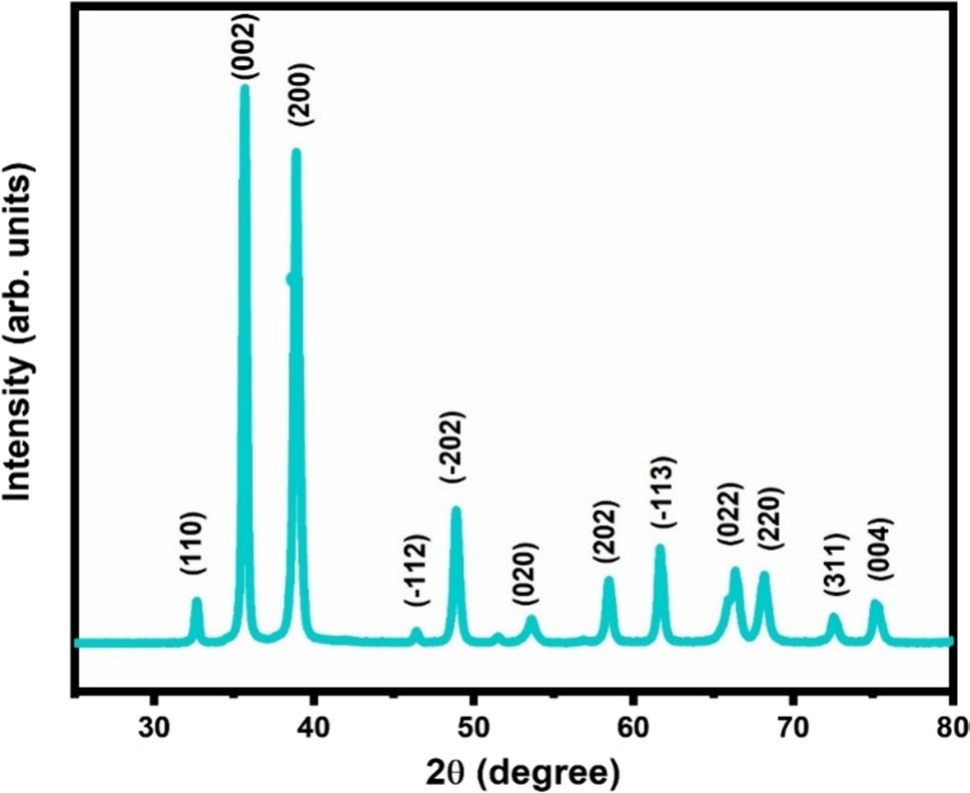
XRD pattern of synthesized CuO sample

With the application of XRD technique, the crystal structure of the synthesised CuO was investigated. The XRD pattern as displayed in Fig. 4 matched with JCPDS card no. 00-002-1041 and corresponds to the monoclinic crystal system of CuO. The diffraction peaks at 2θ values of 32.6°, 35.6°, 38.9°, 46.4° 48.9°, 53.6°, 58.4°, 61.7°, 66.2°, 68.1°, 72.5° and 75.2° belong to the (110), (002), (200), (− 112), (− 202), (020), (202), (− 113), (022), (220), (311) and (004) planes, respectively. Highly intense and sharp peaks of the XRD spectrum show that synthesized material has good crystallinity. Some important parameters like crystallite size, microstrain, interplanar spacing (d spacing) derived from XRD data are listed in Table 1.

**Table 1:** Some structural parameters derived from XRD analysis

S. no	Peak position 2θ (°)	Plane	FWHM β (°)	Crystallite size D (nm)	Microstrain × 10^−3^	D spacing (Å)
1	32.6727	110	0.30296	27.34	4.51	2.70
2	35.67588	002	0.3156	26.46	4.28	2.48
3	38.92196	200	0.454	18.57	5.61	2.27
4	46.42775	− 112	0.34479	25.09	3.51	1.90
5	48.93424	− 202	0.44512	19.62	4.27	1.80
6	53.61722	020	0.69302	12.85	5.98	1.65
7	58.48362	202	0.46755	19.48	3.64	1.51
8	61.7049	− 113	0.43379	21.34	3.17	1.43
9	66.28713	022	0.9847	9.64	6.58	1.33
10	68.16893	220	0.64996	14.76	4.19	1.30
11	72.59152	311	0.58287	16.92	3.46	1.22
12	75.24386	004	0.59947	16.74	3.39	1.17

The average crystallite size (D) of the synthesised sample was calculated from the Debye Scherrer equation,

2 $$D = \frac{K\lambda\!\!\!^{-}}{{\beta cos\theta}}$$

where k is the shape constant, ƛ is the wavelength of the X-ray employed, β is full width at half maxima and θ is the diffraction angle [37] and it was 19.06 nm. The lattice constants a = 4.54 Å, b = 3.30 Å and c = 4.96 Å was calculated from (200), (020) and (002) planes, respectively and obtained values of lattice constants were slightly less than the standard JCPDS card (00-002-1041).

**Fig. 5:**
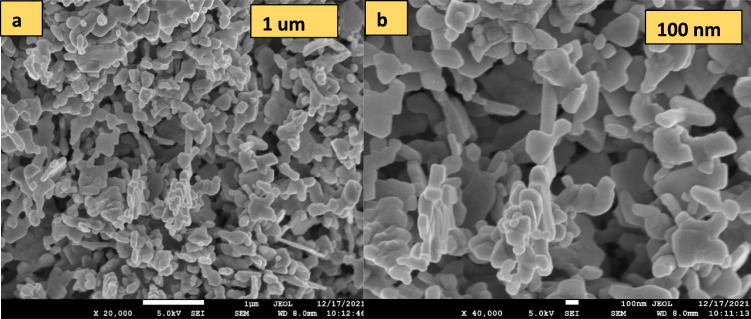
FE-SEM micrographs of CuO samples at different magnifications

To analyse the morphology of the synthesized sample, FE-SEM was performed at various magnifications as displayed in the Fig. 5a, b. Figure 5a shows similar types of structures having a uniform growth at lower magnifications. The Image of higher magnification (Fig. 5b) displayed particle like morphology.

**Fig. 6:**
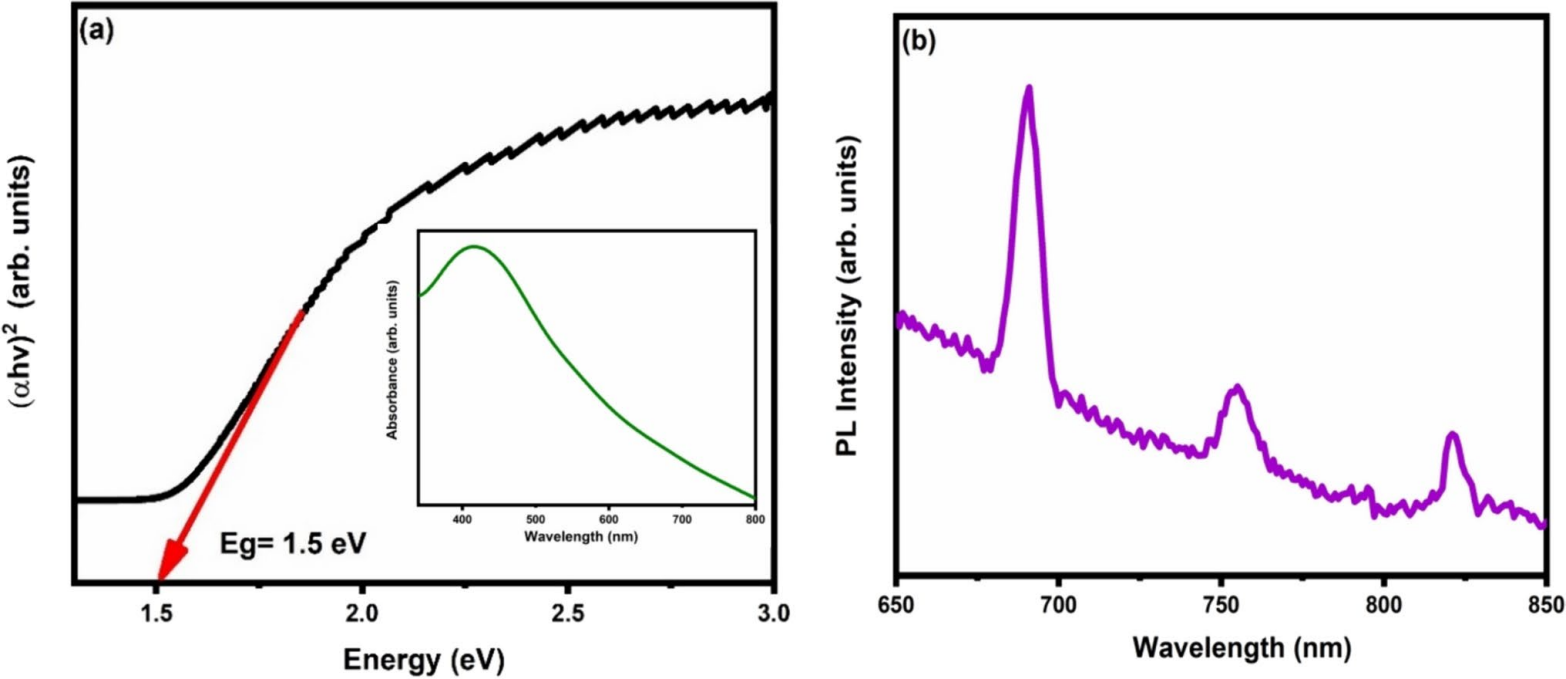
**a** Tauc plot and UV–visible absorption spectrum (inset) **b** photoluminescence spectrum of CuO sample

The band gap and light absorption of the synthesised sample were investigated employing the ultraviolet–visible spectroscopy. The synthesized material's UV–Vis absorption spectrum is depicted in inset of Fig. 6a. The sample shows a large and broad absorption maximum in the visible region. For calculating the band gap from absorption data, Tauc’s equation was used-

3 $$\alpha hv = A\left( {hv - E_{g} } \right)^{1/2}$$

where A is a constant, v is the frequency, h is Planck's constant, α is the absorption constant and Eg is the band gap [38]. The direct band gap of the CuO was determined to be 1.5 eV from the Tauc's plot (Fig. 6a) [39].

In order to investigates the emission spectrum of the material, photoluminescence spectroscopy was used. Emission spectra was recorded using an excitation wavelength of 460 nm in the spectral range of 650 to 850 nm (Fig. 6b). Three emission peaks at 690 nm, 755 nm and 821 nm was observed. Red–orange emission observed at 690 nm was due to the interstitial defect, peak at 755 nm represents a red emission due to the electron–hole pair recombination at the oxygen vacancies [40] and a peak due to recombination of bound excitons was present at 820 nm [24].

**Fig. 7:**
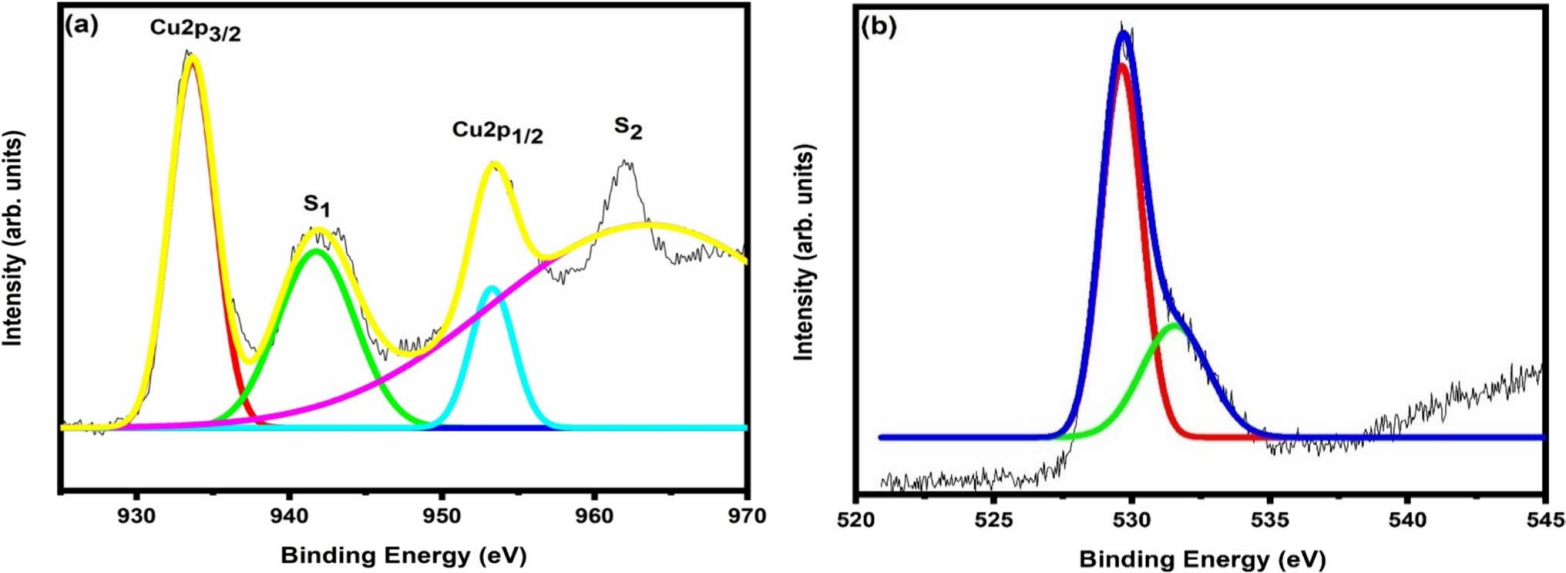
**a** XPS spectrum of the CuO sample for Cu2p peaks **b** O1s peaks

For the investigation of the chemical composition and valence states of the synthesized CuO sample, XPS analysis was performed. Gaussian-fitted XPS spectrum corresponding to Cu2p and O1s are shown in Fig. 7. In Fig. 7a, the peak at 933.54 eV represents the Cu2p_3/2_ which has a satellite peak at 942.20 eV, while the peak at 953.35 eV corresponds to the Cu2p_1/2_ with a satellite peak at 963.68 eV. The location of Cu2p peaks confirms that Cu species in the synthesized sample have + 2 oxidation state. Figure 7b shows O1s peaks at 529.70 and 531.50 eV, which are associated with oxygen ions adsorbed on the surface of CuO and the O^2−^ ion in the lattice of CuO respectively [41]. So, the XPS results confirm that the synthesized material is pure CuO.

**Fig. 8:**
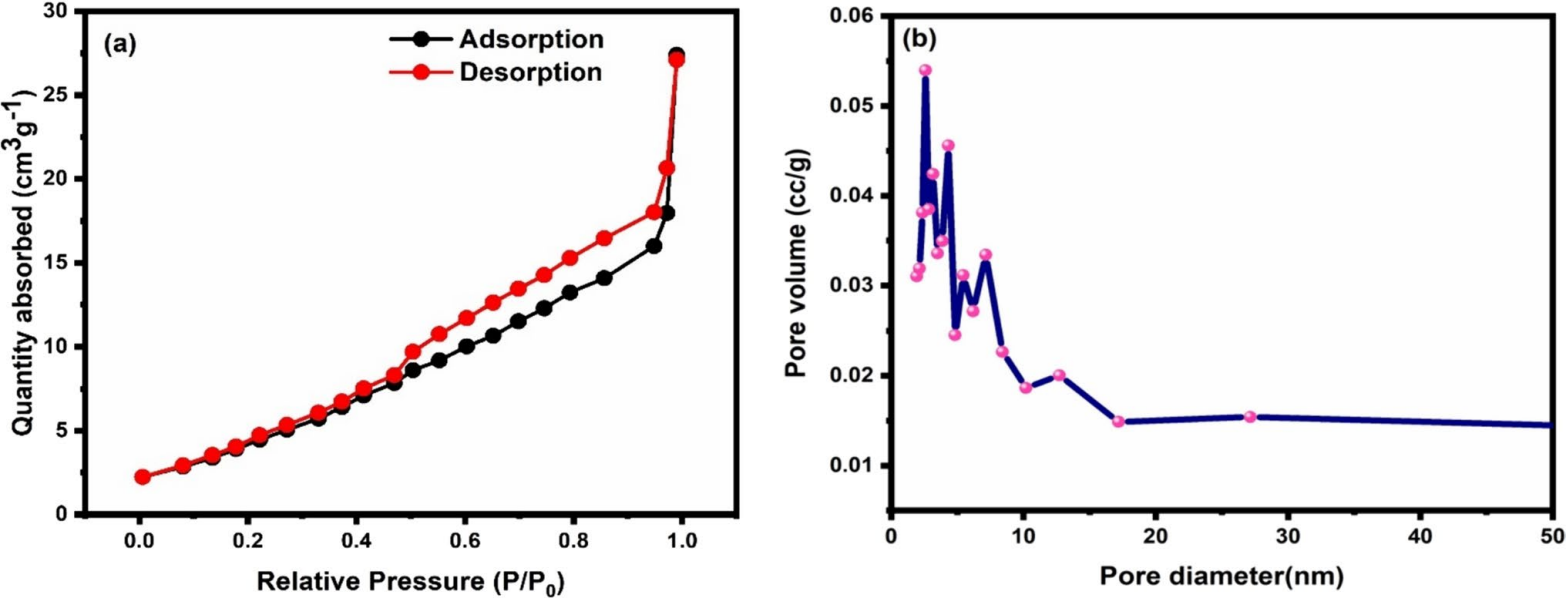
**a** N_2_ adsorption–desorption isotherm **b** pore size distribution of the CuO sample

For studying the surface properties of sample, Brunauer–Emmett–Teller (BET) analysis was done. Figure 8a shows the nitrogen adsorption–desorption isotherm and for low relative pressure that is up to 0.5, a linear region is obtained. The value of surface area as calculated from this linear region comes out to be 20.510 m^2^/g. Figure 8b shows the pore size distribution of the CuO, from which it can be concluded that pore size lies below 30 nm confirming the mesoporous nature of the synthesized sample [42]. The pore diameter of CuO was found equal to 2.595 nm and pore volume equal to 0.041 cc/g using the Barrett-Joyner-Halenda (BJH) method.

### Gas sensing properties

**Fig. 9:**
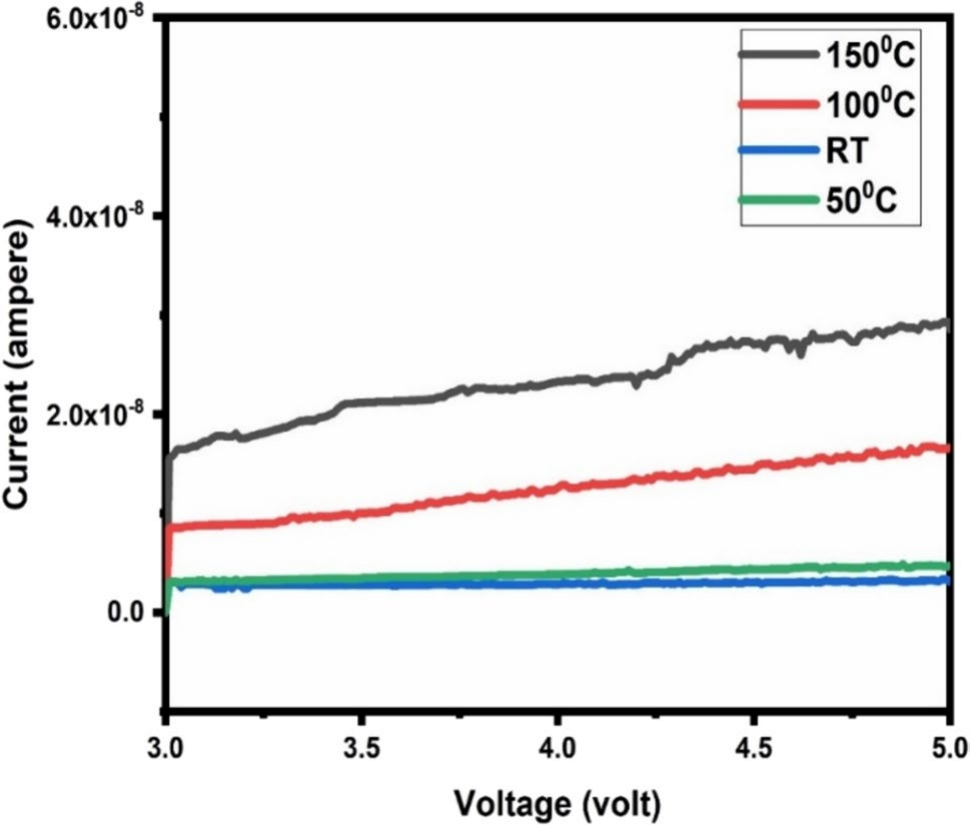
The variation of current with applied voltage for the film at different operating temperature

**Fig. 10:**
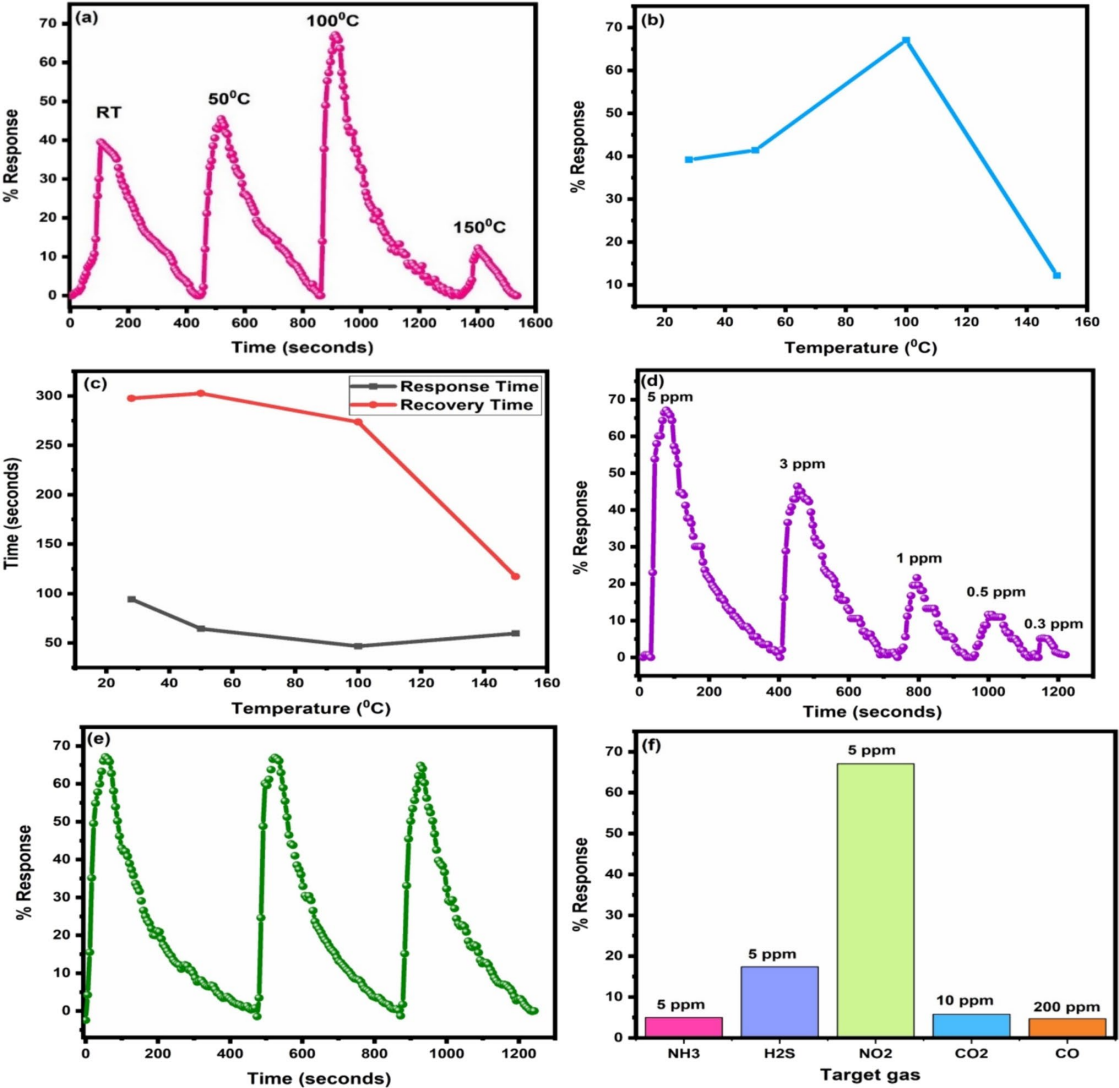
**a** Transient response curve for 5 ppm of NO_2_ gas at different temperatures **b** variation of % response with temperature **c** variation of response and recovery times with temperature **d** transient response curve for different concentrations of NO_2_ gas at 100 °C **e** transient response curve for three successive cycles of 5 ppm NO_2_ gas at 100 °C **f** selectivity study of CuO sensor for 5 ppm concentration at 100 °C

Gas sensing is temperature-dependent so to find the optimal temperature, the response of material to 5 ppm of NO_2_ was checked at room temperature, 50 °C, 100 °C and 150 °C. Prior to the gas injection, variation of current of the film with voltage was checked. Figure 9 shows the IV characteristics of the film at different temperatures from which it can be concluded that the film shows an ohomic behaviour. The transient response curves at room temperature, 50 °C, 100 °C and 150 °C are shown in Fig. 10a. The variation in percentage response with operating temperature is illustrated in Fig. 10b. The highest value of response was noted at 100 °C. So, 100 °C is the optimal temperature of the synthesized sample. Oxygen ions adsorbed on the sensing material’s surface play a significant role in the sensing of NO_2_ gas. At different temperatures, surface of CuO may contain O^2−^, O^−^, or O_2_^−^ ions due to the adsorption of air. Due to the lack of activation energy at lower temperatures, such as 50 °C and room temperature, there is poor gas adsorption [43]. Adsorption process dominated by desorption causes poor response at high temperatures (more than or equivalent to 150 °C). The striking of the adsorption and desorption process is analogues at intermediate temperature, resulting in maximum response towards the gas [44]. The response and recovery times of the sample at different temperatures are shown in Fig. 10c. At room temperature, 50 °C, 100 °C and 150 °C, the material's response times were 94.27 s, 64.39 s, 46.8 s and 59.7 s, respectively. The corresponding recovery times at different temperatures were 117.3 s, 273.6 s, 297.68 s and 302.64 s. At low temperature, response times were long, but at optimum temperature, it was short. Recovery time was almost the same at all temperatures except at 150 °C. At 150 °C, recovery was fast but the % response was less because of the quick gas desorption. After optimizing the temperature, the film was tested for varying concentrations of NO_2_ gas (Fig. 10d). The response was checked for 5, 3, 1, 0.5 and 0.3 ppm of sensing gas. The % response was observed equal to 67.1%, 46.4%, 21.6%, 11.67% and 5.2% for 5, 3, 1, 0.5 and 0.3 ppm of NO_2_ gas, respectively. So, the synthesized sample was able to detect even 300 ppb of gas with a percentage response value of 5.2%. Additional file 1: Fig. S1 shows the variation of current with time for different concentrations of NO2 gas (Online Resource). The % response decreased as the gas concentration decreased. It may be due to the fact that the number of gas molecules adsorbed on the surface of material directly affects the response and at lower ppm values, there are fewer gas molecules available for adsorption.

**Fig. 11:**
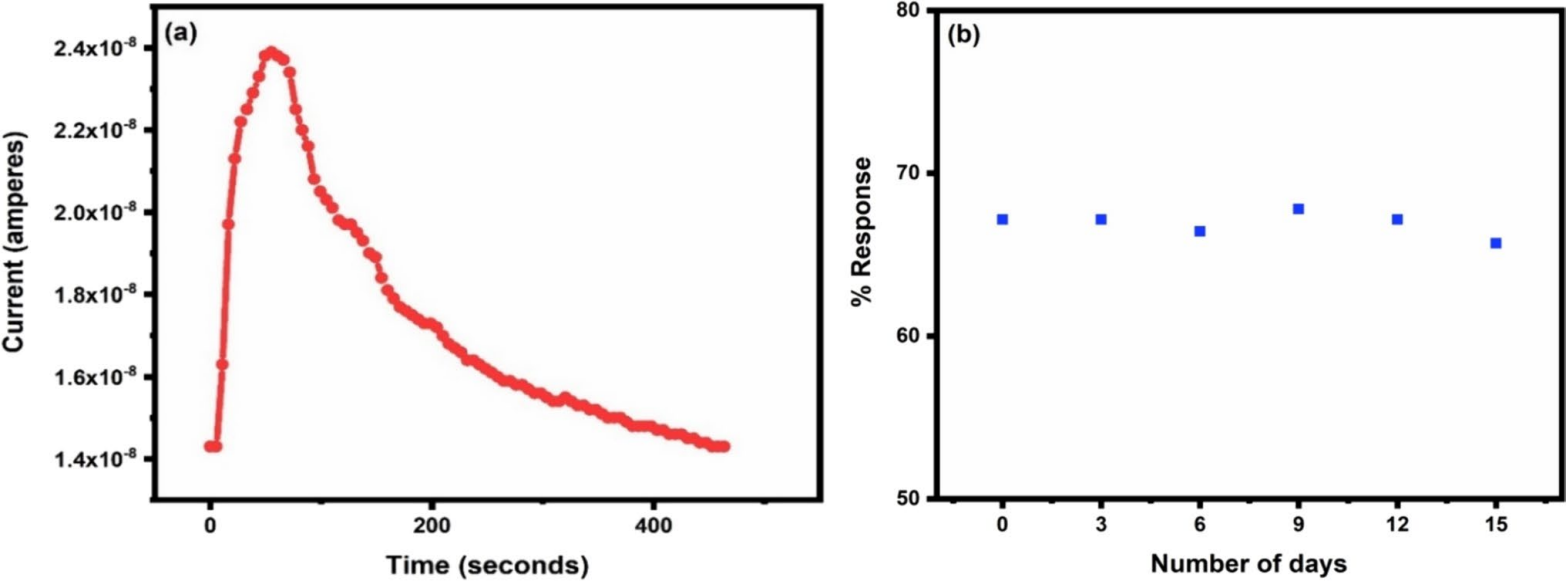
**a** Variation of current with time for 5 ppm of NO_2_ gas at 100 °C **b** stability curve of CuO for 5 ppm of NO_2_ gas at 100 °C

To test the material’s repeatability, three successive cycles of 5 ppm of NO_2_ gas at the optimized temperature were recorded (Fig. 10e). The sensor showed nearly same value of % response after repeated exposure and gas removal. Figure 10f shows the selectivity graph with 5 ppm of NO_2_, 200 ppm of CO, 10 ppm of CO_2_, 5 ppm of H_2_S (Additional file 1: Fig. S2 and S3 in Online Resource) and 5 ppm of NH_3_ gases. At the optimized temperature, the sample shows 67.1%, 4.7%, 5.8%, 17.43% and 5% response towards 5 ppm of NO_2_, 200 ppm of CO, 10 ppm of CO_2_, 5 ppm of H_2_S and 5 ppm of NH_3_ gases respectively. It can be inferred from the selectivity graph that the sample shows maximum response and hence is highly selective for NO_2_ gas. The underlying reason for this occurrence may be the fast interaction occurring between the copper oxide surface and the gas being analysed. A comparison of the performance of various CuO sensors reported in literature and the present work is shown in Table 2. Figure 11a shows the variation of current with time when CuO was exposed to 5 ppm of NO_2_ gas at optimal temperature. To check the stability of sensor, response for 5 ppm of NO_2_ gas was checked for 15 days taking time interval of three days. Figure 11b represents the stability curve which shows nearly similar values of % response except some minor variations indicating good stability of the sensor. The results conclude that the synthesized material has exceptional sensing characteristics, including high % response at a lower operating temperature for low concentration of NO_2_ gas, quick response and recovery times, a low detection limit, good selectivity, excellent stability and repeatability. As a result, it is an ideal candidate for detecting extremely low concentrations of NO_2_ gas.

**Table 2:** Comparison of the present study with similar other works

S. no	Sensing material	Method of preparation	Concentration (ppm)	Operating temperature (°C)	Response	Detection limit (ppm)	Reference
1	CuO	Thermal evaporation	100	150	76%	1 ppm	[45]
2	CuO	Hydrothermal	20	Room temperature	14.5*	10	[46]
3	CuO–Co_3_O_4_	Hydrothermal	10	160	37.86%		[47]
4	CuO–ZnO	Thermal oxidation	100	250	4.1^#^		[48]
5	CuO	Chemical reduction	100	250	22%		[49]
6	Pd–CuO	Sol–gel	5	125	38.9%	0.82	[50]
7	CuO	Spray pyrolysis	100	200	56%	5	[51]
8	CuO–ZnO	Precipitation	100	Room temperature	36.7%	5	[52]
9	CuO	Precipitation	5	100	67.1%	0.3	This work

*R_bg_/R_tg_

^#^R_g_/R_a_

#### Sensing mechanism

The sensing mechanism relies on a charge transfer process, which is regulated by the adsorption and desorption of oxygen ions on the sensing material's surface [53, 54]. Copper oxide is a p-type semiconductor and it is highly adsorbent to oxygen. Current in the material changes due to the change of adsorbed oxygen species in oxygen ion. In the presence of oxidizing gases, the current in p-type semiconductors increases, while it decreases in reducing gases. The current in the sample at a particular temperature is determined by the presence of majority charge carriers, which in the case of copper oxide are holes. Oxygen molecules present in the air adsorbs electron from the conduction band of CuO and coverts to O^2−^, O^−^ or O_2_^−^. Due to the adsorption of electrons by oxygen, more holes are produced in copper oxide resulting in the formation of hole accumulation layer. Type of oxygen ion present depends on the operating temperature [55].

4 $$O_{2 } \left( {air} \right) \to O_{2} \left( {adsorbed} \right)$$

5 $$O_{2} \left( {adsorbed} \right) + e^{ - } \to O_{2}^{ - } \left( {adsorbed} \right)$$

6 $$O_{2}^{ - } \left( {adsorbed} \right) + e^{ - } \to 2O^{ - } \left( {adsorbed} \right)$$

**Fig. 12:**
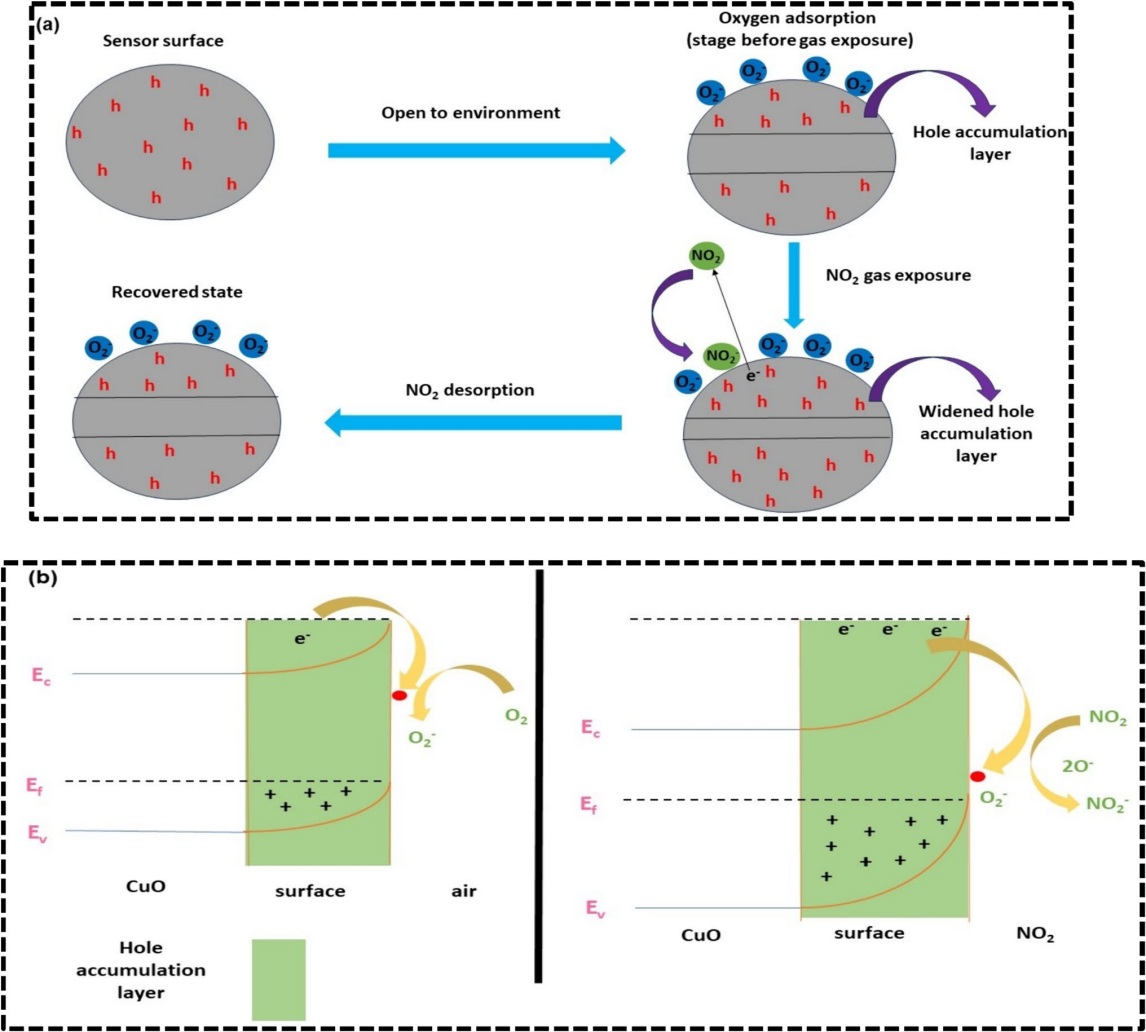
**a** Mechanism for the sensing of NO_2_ gas on the surface of CuO **b** energy band diagram for NO_2_ sensing by CuO

For the synthesized sample at optimal temperature that is 100 °C, oxygen is adsorbed as O_2_^−^ ion as shown in Eqs. (4)–(6). As a result of chemical interaction between copper oxide and NO_2_ gas, concentration of holes can be changed. NO_2_ is an oxidising gas, so it takes electrons from the sensing material to form NO_2_^−^ as shown in Eqs. (7)–(10). This phenomenon causes further cause electron depletion and a significant increase in hole concentration is there due to which the hole accumulation layer gets widened. So, after the adsorption of the NO_2_ gas current in the material increases [56, 57]. The NO_2_^−^ get evaporated when the gas flow is stopped and captured electrons are released back to CuO. These electrons recombine with the holes, resulting in resulting in decreased hole density and, hence current [49]. The schematic diagram for sensing the NO_2_ gas by CuO is shown in Fig. 12a, b.

7 $$NO_{2 } \left( {gas} \right) \to NO_{2} \left( {adsorbed} \right)$$

8 $$NO_{2} \left( {adsorbed} \right) + e^{ - } \to NO_{2}^{ - } \left( {adsorbed} \right)$$

9 $$NO_{2} \left( {adsorbed} \right) + O_{2}^{ - } + 2e^{ - } \to NO_{2}^{ - } \left( {adsorbed} \right) + 2O^{ - } \left( {adsorbed} \right)$$

10 $$NO_{2}^{ - } \left( {adsorbed} \right) + 2O^{ - } \left( {adsorbed} \right) + e^{ - } \to NO_{2 } \left( {gas} \right) + 2O^{2 - } \left( {adsorbed} \right)$$

The mechanism of adsorption of oxygen species on the CuO surface can be of two types that is chemisorption or physisorption. Physisorption is the process that happens at low temperatures while the chemisorption process occurs at high temperatures. In the present study, CuO shows a good response at low temperature for NO_2_ gas indicating that the bond between surface of CuO and oxygen ion is enough for sensing the NO_2_ gas. With the increase in temperature, the bond between CuO and oxygen ions becomes stronger and different oxygen ions become active (Eqs. 4–6). O_2_ (air) represents the ambient atmosphere which accepts electrons from the conduction band of CuO. Adsorption of NO_2_ gas takes place on the oxygen ions on the surface of CuO increasing hole density. An increase in the number of holes increases the width of the hole accumulation layer and hence increases the current.

## Conclusion

The synthesis of CuO nanostructures utilized a precipitation method, followed by various characterization techniques. X-ray diffraction (XRD) outcomes validate the synthesized material's excellent crystallinity, revealing a crystallite size of 19.06 nm. The particle-like morphology of the synthesized material is revealed by FE-SEM analysis. The sample exhibits a wide absorption peak in the visible spectrum, indicating a band gap of 1.5 eV and photoluminescence spectroscopy reveals the presence of defects attributed to bound excitons, oxygen vacancies and interstitial oxygen. XPS analysis conclusively demonstrates the exclusive presence of Cu in its + 2-oxidation state, confirming the pristine nature of the synthesized material as pure CuO. BET analysis confirms the mesoporous nature and high surface area of the CuO sample.

In the gas sensing measurements, the fabricated film exhibited its peak performance at an optimal temperature of 100 °C. The material demonstrated the capability to detect NO_2_ gas at concentrations as minimal as 300 ppb, yielding a 67.1% response for 5 ppm gas. Therefore, the synthesized material exhibits exceptional sensing attributes, encompassing high percentage response, detection limits in the sub-parts per million range, good selectivity, stability and repeatability. Thus, the present study enables the creation of a cost-effective and low-temperature operational sensing material, suitable for selectively detecting exceedingly low concentrations of NO_2_ gas.

## Supplementary Information

**Additional file 1.**

## Data Availability

The datasets generated during and/or analysed during the current study are available from the corresponding author on reasonable request.
