# Supplementary material for: Low ppm NO2 detection through advanced ultrasensitive copper oxide gas sensor
Source: Discov Nano. 2024 Jun 24;19(1):107. doi: 10.1186/s11671-024-04039-z (PMC11557803; doi:10.1186/s11671-024-04039-z)
Supplement: Supplementary file 1 — Additional file 1. [file 11671_2024_4039_MOESM1_ESM.docx]

**Supplementary Information**

The analysis has been done in presence of H_2_S gas, a response of 17.43% was recorded for 5 ppm of H_2_S gas at 100^0^C with response and recovery times of 119.24 seconds and 377.89 seconds respectively. Current vs time curve and response curve for H_2_S gas has been shown in Fig. S1 and Fig. S2.

The % Response for H_2_S gas was much less as compared to NO_2_ gas, which signifies the selectivity of the synthesized sensor towards NO_2_ and also response and recovery times were also much larger. The Current versus time graph for different concentration of NO_2_ gas has been demonstrated in Fig. S3


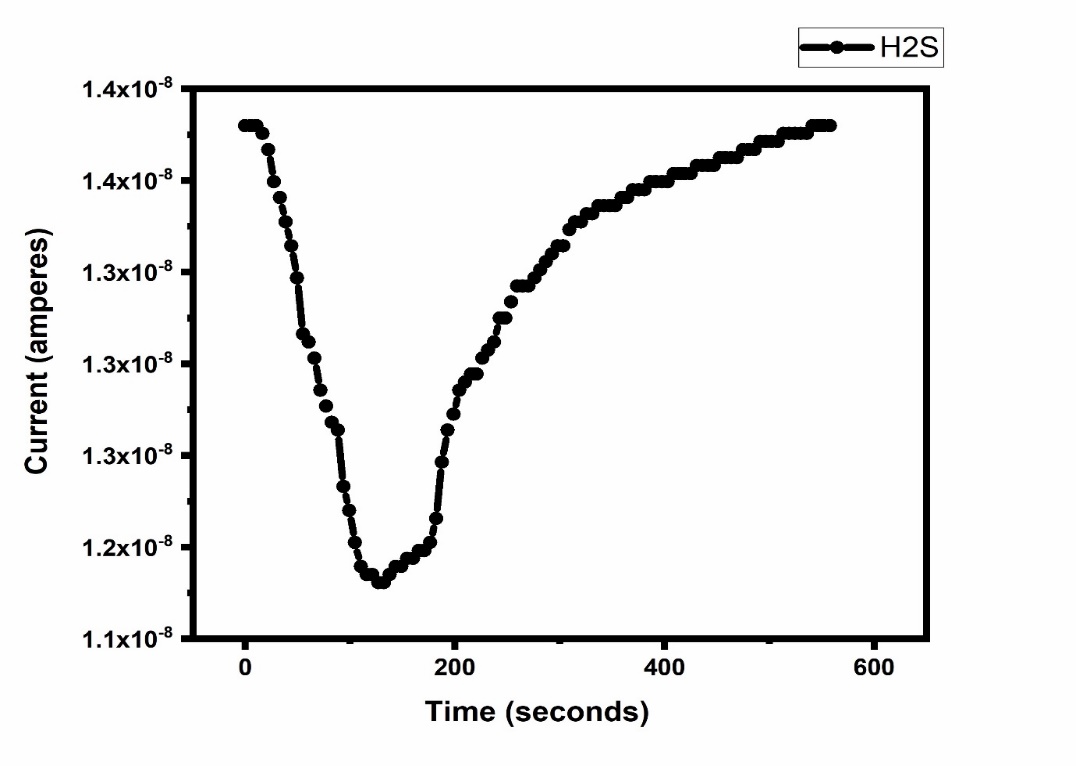


**Fig. S1** Variation of current with time for curve for 5 ppm of H_2_S gas at 100^ο^C


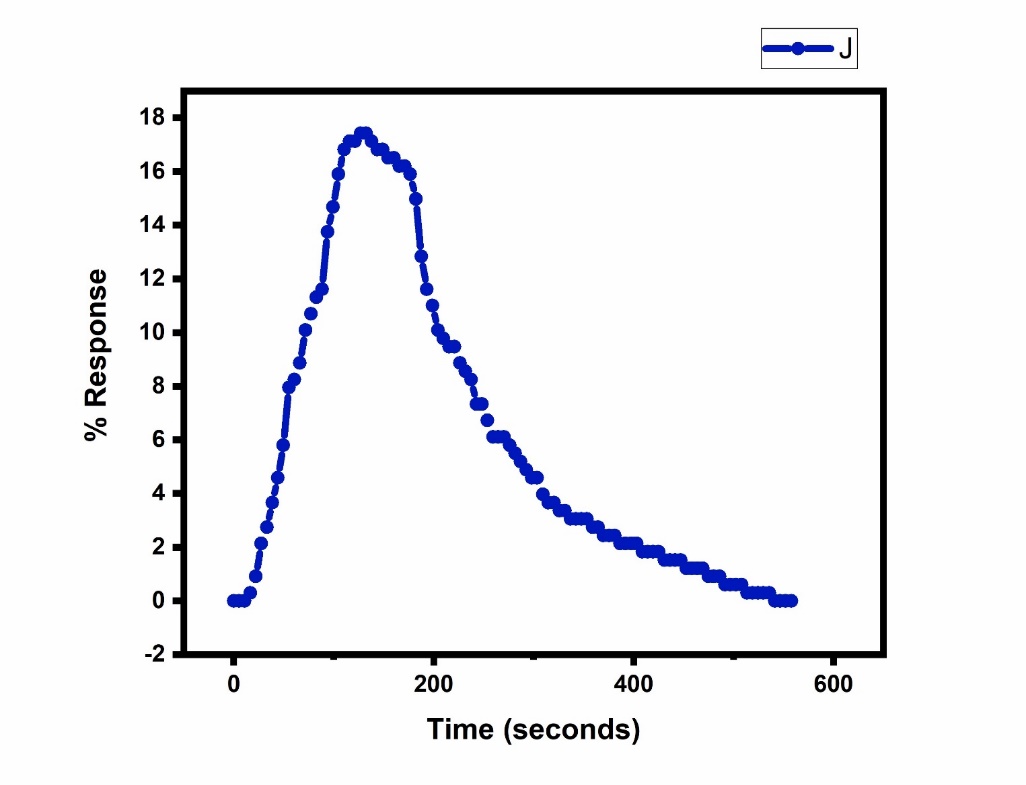


**Fig. S2** Transient response curve for 5 ppm of H_2_S gas at 100^ο^C


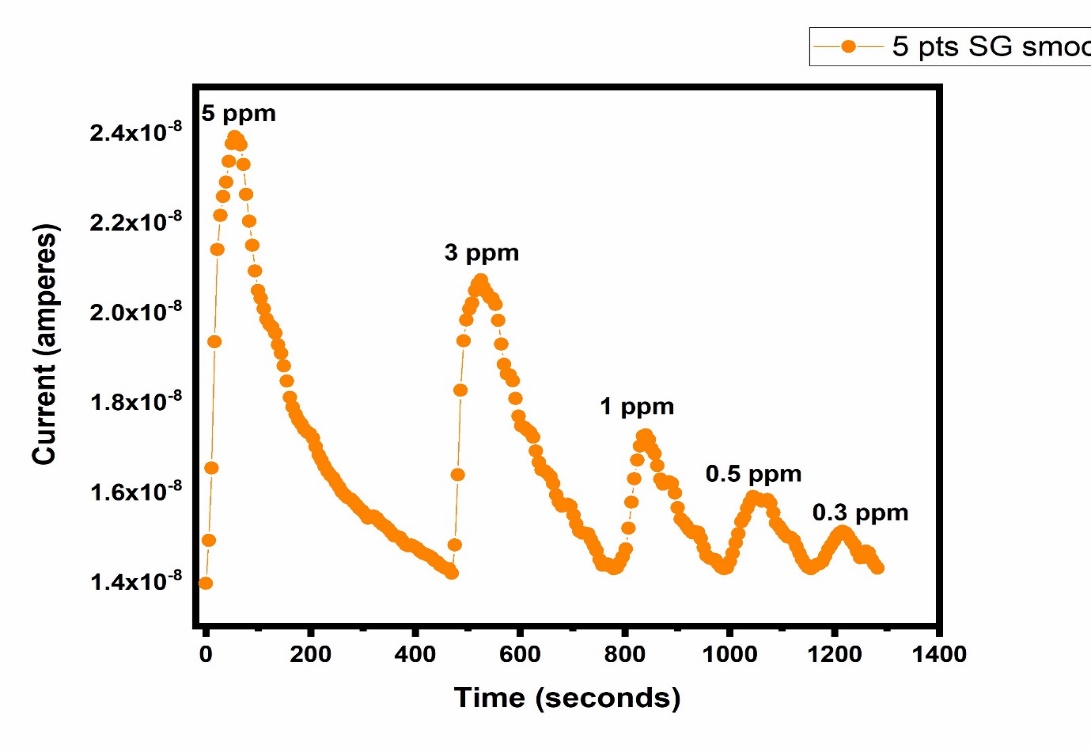


**Fig. S3** Variation of current with time for different concentrations of NO_2_ gas at 100^ο^C
